# Cost-Effectiveness of Home-Based Self-Sampling vs Clinician Sampling for Anal Precancer Screening

**DOI:** 10.1001/jamanetworkopen.2025.52220

**Published:** 2026-01-05

**Authors:** Haluk Damgacioglu, Timothy L. McAuliffe, Timothy J. Ridolfi, Elizabeth Chiao, Maria E. Fernandez, Vanessa Schick, Jennifer S. Smith, Bridgett Brzezinski, Jenna Nitkowski, Ashish A. Deshmukh, Alan G. Nyitray

**Affiliations:** 1 Department of Public Health Sciences, Medical University of South Carolina, Charleston; 2 Cancer Prevention & Control Program, Hollings Cancer Center, Medical University of South Carolina, Charleston; 3 Center for Community Health and Intervention Research, Medical College of Wisconsin, Milwaukee; 4 Department of Surgery, Medical College of Wisconsin, Milwaukee; 5 MD Anderson Cancer Center, The University of Texas, Houston; 6 Department of Health Promotion and Behavioral Sciences, The University of Texas Health Science Center at Houston School of Public Health, Houston; 7 Department of Management, Policy and Community Health, The University of Texas Health Science Center at Houston School of Public Health, Houston; 8 Gillings School of Global Public Health, University of North Carolina at Chapel Hill, Chapel Hill; 9 Center for Cancer Discovery, Medical College of Wisconsin, Milwaukee

## Abstract

**Importance:**

Anal cancer screening is recommended for high-risk populations, particularly sexual and gender minority (SGM) individuals. However, the cost-effectiveness of home-based self-sampling in increasing anal cancer screening uptake has not yet been evaluated in the US.

**Objective:**

To evaluate the cost-effectiveness of home-based anal self-sampling compared with clinic-based screening among SGM individuals.

**Design, Setting, and Participants:**

This economic evaluation used data from a 2-group randomized clinical trial conducted in Milwaukee, Wisconsin, from January 2020 to August 2022, enrolling SGM individuals aged 25 years or older. Participants were randomized to home-based self-sampling or clinic-based screening. Costs for home-based screening were obtained from the trial, and clinic-based costs were sourced from the Medicare reimbursement schedule. Travel and time costs were derived on the basis of participant self-reports. The analysis was performed between February and October 2025.

**Intervention:**

Participants in the home-based screening group received self-sampling supplies and instructions, and those in the clinic-based screening group were instructed to visit a clinic for anal cancer screening.

**Main Outcomes and Measures:**

The primary outcome was the incremental cost-effectiveness ratio (ICER), measured as the additional cost needed to increase screening participation by one person. The 95% CIs for the ICERs were estimated using a bootstrap method with 1000 iterations. Net benefit regression and cost-effectiveness acceptability curves were used to assess the likelihood of cost-effectiveness across different willingness-to-pay (WTP) thresholds.

**Results:**

The study included 240 SGM individuals (227 with gender identity as a man [95%]; median [IQR] age, 46 [33 to 57] years), of whom 65 (27%) had HIV. The cost per participant was $64.18 for home-based screening and $60.40 for clinic-based screening from a societal perspective, and $61.91 for home-based screening and $42.06 for clinic-based screening from a health care payer perspective. Home-based screening was associated with increased screening participation vs clinic-based screening (107 participants [89.2%] vs 89 participants [74.2%]). The ICER per additional screened participant was $25.19 (95% CI, −$27.66 to $104.60) for the societal perspective and $132.36 (95% CI, $74.54 to $402.20) for the health care payer perspective. Home-based screening had a 49.6% probability of being cost-effective at a WTP of $25, 99.99% at a WTP of $100 (societal perspective), and 90.9% at a WTP of $200 (health care payer perspective). The ICERs for home-based screening compared with clinic-based screening were highly sensitive to screening participation rates.

**Conclusions and Relevance:**

The findings of this economic analysis suggest that home-based anal cancer screening is a cost-effective approach to increasing screening participation among SGM individuals. Home-based screening may serve as a valuable and efficient tool for expanding screening rates.

## Key Points

**Question:**

What is the cost-effectiveness of home-based vs clinic-based anal cancer screening in increasing screening uptake among sexual and gender minority (SGM) groups in the US?

**Findings:**

In this economic evaluation of a randomized clinical trial involving 240 SGM men and transgender women, the incremental cost-effectiveness ratio for increased screening uptake was $132.36 from the health care payer perspective and $25.19 from the societal perspective per additional completed screening.

**Meaning:**

These findings suggest that home-based anal cancer screening is a cost-effective strategy for increasing screening participation among SGM individuals, particularly from a societal perspective, as it reduces travel and time-related costs.

## Teaser

This economic evaluation examines the cost-effectiveness of home-based anal self-sampling compared with clinic-based screening among sexual and gender minority individuals.

## Introduction

Squamous cell carcinoma of the anus is the most common (over 90%) subtype of anal cancer caused by human papillomavirus (HPV) infection.^1^ Although it is relatively rare in the general population,^2^ squamous cell carcinoma of the anus disproportionately affects men who have sex with men (MSM) with HIV (85 cases per 100 000 MSM; 32 cases per 100 000 non-MSM with HIV), MSM without HIV (16 cases per 100 000 persons aged ≥30 years), solid-organ transplant recipients (13 cases per 100 000 persons), and women with a history of vulvar cancer (48 cases per 100 000) or precancer (42 cases per 100 000).^3,4^

Squamous cell carcinoma of the anus is preceded by a detectable anal precancer, high-grade squamous intraepithelial lesions (HSIL). The recent Anal Cancer–HSIL Outcomes Research study demonstrated that treating anal HSIL significantly reduces the risk of anal cancer in individuals with HIV.^5^ This new evidence led the US Department of Health and Human Services (HHS), the International Anal Neoplasia Society, and several other international professional organizations to recommend anal cancer screening for high-risk individuals.^6,7,8^

Because of similarities in risk factors and screening practices, anal cancer screening may benefit from lessons learned in cervical cancer programs. Cervicovaginal self-sampling has been shown to detect HPV DNA effectively and has been introduced in some countries to address barriers to screening uptake, which are influenced by factors such as access to care, systemic racism, stigma, and cultural norms.^9,10,11,12^ Similarly, anal cancer screening may encounter barriers like stigma and embarrassment related to anogenital examinations, sexually transmitted infections, anal sex, and anal cancer.^13,14,15,16^

The Prevent Anal Cancer (PAC) Self-Swab Study assessed screening engagement and the adequacy of specimens for HPV DNA testing collected in home-based anal self-sampling and clinic-based clinician sampling.^17^ The study observed that home-based anal self-sampling may enhance engagement and offer a more equitable alternative compared with standard clinic-based screening.^17^ A detailed economic analysis of home-based screening is critical for a successful implementation of a self-sampling program. However, to date, no study has evaluated the cost-effectiveness of self-sampling for anal cancer screening. This study aims to address this gap by assessing resource utilization and conducting an economic evaluation of home-based anal self-sampling vs clinic-based screening approaches.

## Methods

In this economic evaluation, we used data from a prospective, 2-group, randomized clinical trial, the PAC Self-Swab Study in Milwaukee, Wisconsin, which recruited sexual and gender minority (SGM) individuals from January 2020 to August 2022. Eligibility criteria included individuals aged years 25 or older who identified as SGM and had engaged in sex with men in the past 5 years. Exclusions were made for individuals with a prior anal cancer diagnosis, those taking anticoagulants, or individuals with conditions such as hemophilia or thrombocytopenia, as well as those living outside of the Milwaukee metropolitan area. Detailed descriptions of the trial have been published previously,^17^ and the trial was registered at ClinicalTrials.gov (NCT04090060). The analysis was performed between February and October 2025. Study procedures were approved by the Medical College of Wisconsin Human Research Protections Program. All individuals enrolled provided informed consent to participate in the study. This study follows the Consolidated Health Economic Evaluation Reporting Standards (CHEERS) reporting guidelines.

### Intervention and Effect Estimation

During the online consent session, study staff explained the study procedures, discussed anal cancer screening risks, and addressed concerns related to HPV DNA screening. Participants were informed they would be asked to screen at the beginning of the study and again 1 year later. Baseline HPV test results were not shared with the participants. However, notifications were sent if persistent high-risk HPV was detected after 1 year. Participants were randomly assigned (1:1) to either the home or the clinic group. Randomization was not blinded, whereas specimen processing was blinded.

Home-group participants were mailed a PAC Pack with self-sampling supplies and instructions. The PAC Pack included a nylon-flocked swab (Copan Diagnostics), a vial of Standardized Transport Medium (Digene Corporation), gloves, and an ambient temperature recording device. Participants followed self-sampling instructions and returned the PAC Pack to the Medical College of Wisconsin tissue bank via mail. After home sampling, they were also asked to visit a study clinic for a baseline digital anal rectal examination to detect undiagnosed anal cancer. Clinic group participants were instructed to visit 1 of 5 study clinics for clinician-conducted sampling and digital anal rectal examination. Sampling instructions for both lay individuals and clinicians were the same.^18^ For this analysis, we assessed screening participation, with engagement defined as either (1) the return of a PAC Pack to the tissue bank by home-group participants or (2) the completion of a swabbing appointment with a clinician by clinic-group participants.

### Cost Estimation

#### Direct Costs

Direct costs were the expenses associated with screening implementation, including mailing kits for the home-based screening group, and sample processing. In the home-based screening group, costs for materials and mailing were accounted for regardless of whether kits were returned. Processing costs were applied only if a kit was returned to the clinic. The costs for materials, mailing, and processing were derived from the trial data.

In the clinic-based group, costs were incurred only if participants engaged with the screening. This included both material and processing costs. Processing costs were consistent across both screening groups. In addition, we incorporated the cost of office visits, which was obtained from the *Current Procedural Terminology* and the Physician Fee Schedule. Since the procedure did not require specialized interventions, we used the nurse visit category to represent these costs.

#### Time Costs

The questionnaire collected data on the time patients spent traveling to and from the clinic for the clinic-based screening group and to and from the post office or post box for the home-based screening group. On the basis of our trial observations, we estimated the procedure time for home-based screening to be 10 minutes and for clinic-based screening to be 30 minutes. Participants were also asked whether they took time off work. To estimate the time taken off work due to screening, we combined travel time and care time. Thus, the total time costs for participants were the sum of these elements. For those who reported taking time off work, we calculated the cost of their time using the mean hourly wage rate for US workers, which is $34.36 per hour.^19^

#### Travel Costs

Participants were asked questions about their transportation methods, distances traveled, and time spent traveling for the screening. They reported the costs associated with fares for taxis, buses, or other modes of transport. For those who drove cars, we estimated the marginal costs according to reported travel distances using the Internal Revenue Service’s standard mileage rate of 65.5 cents per mile.^20^ For participants using other forms of transportation, such as taxis or public transit, we used the reported fare amounts. Individuals who walked or cycled were considered to have had no transportation costs.

### Statistical Analysis

#### Cost-Effectiveness Analysis

Incremental cost-effectiveness ratios (ICERs) were used to measure the cost per additional person screened by comparing the clinic-based screening with the home-based screening. We calculated ICERs from both the health care sector and societal perspectives. The health care sector perspective included only direct medical costs, while the societal perspective accounted for direct medical costs as well as travel and other time costs. We estimated 95% CIs for the ICERs using a bootstrap method with 1000 iterations.^21^ Net benefit regression and cost-effectiveness acceptability curves were used to assess decision uncertainty, showing the probability that an intervention is cost-effective compared with an alternative over different willingness-to-pay (WTP) levels.^22^

We calculated the net benefit for each individual using the following formula: *NB_i_* = λ*E_i_ − Ci*, where λ represents how much the decision-maker is willing to pay for each unit of effectiveness *E*, and *C* is the cost of delivering the intervention to individual *i*. In the regression model, *NB_i_* is the dependent variable and is expressed as *NB_i_* = β_0_* + *β_1_*I + *ε, where *I* is an indicator variable (1 if the participant was in the home-based screening group and 0 if they were in the clinician sampling group). The coefficient β_1_ indicates the mean difference in net benefit between the home-based screening group and the clinician-based screening group. If β_1_ is positive, the home-based screening is considered cost-effective compared with the clinic-based screening. The 1-sided *P* value for β_1_ provides the probability that the home-based screening is cost-effective compared with the clinic-based screening.^22,23^ We estimated the net benefit regression for various hypothetical values of λ.

#### Sensitivity Analysis

We conducted a comprehensive sensitivity analysis to evaluate the robustness of our cost-effectiveness results. First, we performed a 1-way sensitivity analysis by varying 1 parameter at a time while keeping others constant to assess how changes in individual parameters affected the ICER. Next, we assessed 1 million scenarios with a 0.1% increment by analyzing all possible combinations of effectiveness for home-based self-sampling and clinic-based sampling to determine their combined impact on cost-effectiveness. Finally, we performed a probabilistic sensitivity analysis by simulating 10 000 different combinations of cost and effectiveness parameters. We estimated the distribution of ICERs and evaluated the overall uncertainty in the cost-effectiveness of the screening interventions. All analyses were performed and figures were created using MATLAB software version R2023a (MathWorks).

## Results

The study analyzed data for 240 SGM individuals (227 with gender identity as a man [95%]; median [IQR] age, 46 [33-57] years), of whom 65 (27%) had HIV. Participants were randomized to either home-based screening (120 participants) or clinic-based screening (120 participants) (eTable in Supplement 1). From a societal perspective, the cost per participant was $64.18 for home-based screening and $60.40 for clinic-based screening; from a health care payer perspective, and the cost per participant was $61.91 for home-based screening and $42.06 for clinic-based screening. (Table 1). During the trial, 89.2% of participants in the home-based screening group were screened (107 participants), compared with 74.2% (89 participants) in the clinic-based screening group.^17^ With these levels of engagement, Table 2 presents the ICER values from the cost-effectiveness analysis comparing home-based screening with clinic-based screening, from both societal and health care payer perspectives. From the societal perspective, the incremental cost per additional individual screened was $25.19 (95% CI, −$27.66 to $104.60), and from the health care payer perspective, the ICER was higher at $132.36 (95% CI, $74.54 to $402.20).

**Table 1.** Parameters Used in the Cost-Effectiveness Analysis for Each Screening Strategy

Model parameters	Value	Distribution
Clinic-based screening		
Material cost, $	7.33	Uniform
Office visit cost, $	23.38	Uniform
Sample processing, $	26	Uniform
Travel time, min	39.56	γ
Travel cost, $	10.69	γ
Missed work probability, %	0.337	β
Office visit time, min	30	Uniform
Screening probability, %	74.2	β
Home-based screening		
Material cost, $	22.12	Uniform
Kit distribution cost, $	16.61	Uniform
Sample processing, $	26	Uniform
Travel time, min	7.99	γ
Travel cost, $	2.54	γ
Missed work probability, %	0	β
Test time, min	10	Uniform
Screening probability, %	89.2	β

**Table 2.** Incremental Cost-Effectiveness: Cost Per Additional Individual Screened

Screening strategy	Cost per participant, $	Incremental cost, $	Screening rate, %	Incremental effectiveness	ICER, $
Societal perspective					
Clinic based	60.40	NA	74.2	0.15	25.19 (−27.66 to 104.60)
Home based	64.18	3.78	89.2		
Health care payer perspective					
Clinic based	42.06	NA	74.2	0.15	132.36 (74.54 to 402.20)
Home based	61.91	19.85	89.2		

Abbreviations: ICER, incremental cost-effectiveness ratio; NA, not applicable.

**Figure 1.**
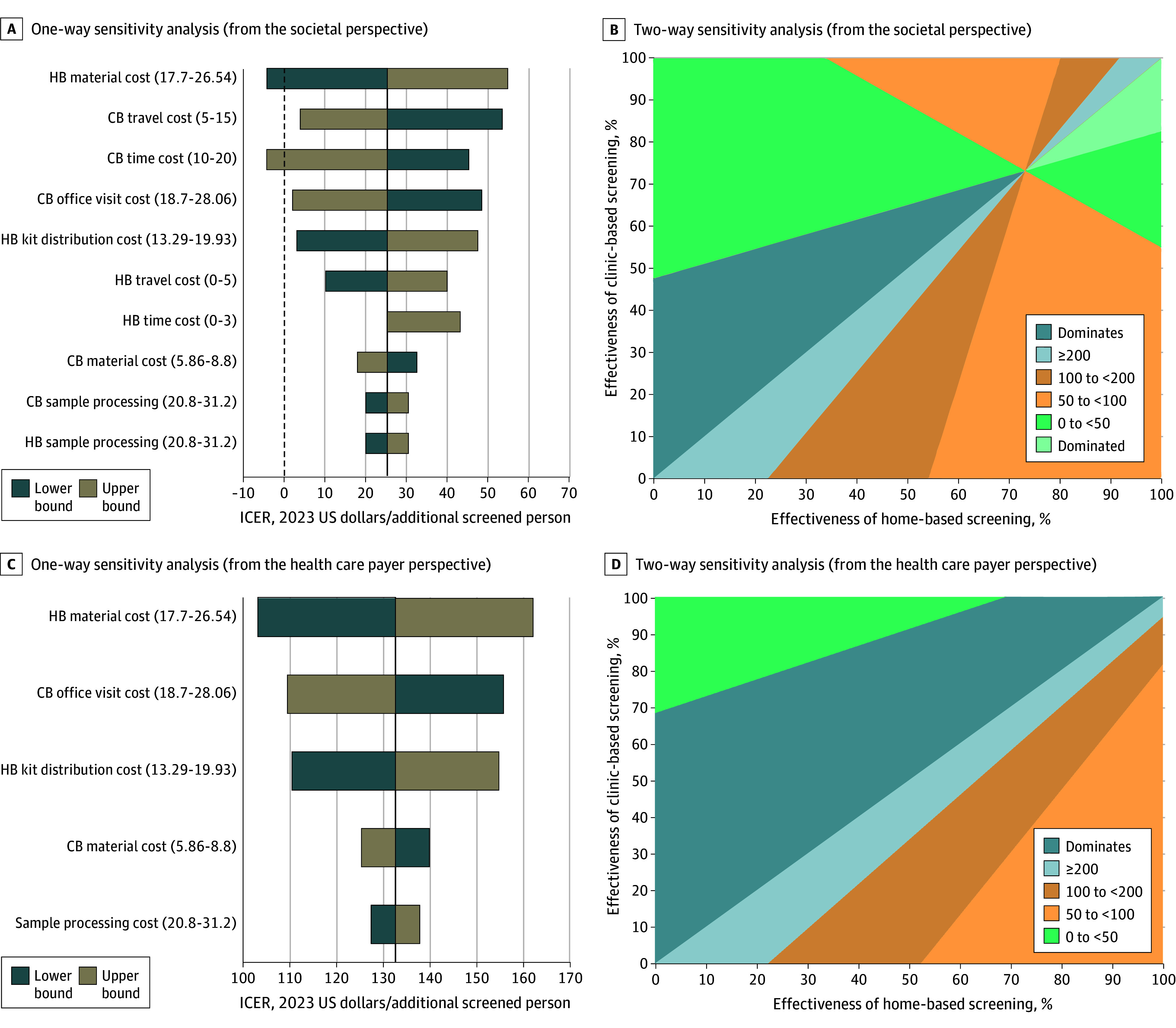
Results of 1-Way and 2-Way Sensitivity Analysis A, One-way sensitivity analysis shows how the cost-effectiveness of home-based (HB) screening compared with clinic-based (CB) screening varies with changes in a single parameter across a range of values for that parameter from the societal perspective. B, Two-way sensitivity analysis shows how the cost-effectiveness of HB screening compared with CB screening changes when varying their screening probability parameters simultaneously, from the societal perspective. C, One-way sensitivity analysis shows how the cost-effectiveness of HB screening compared with CB screening varies with changes in a single parameter across a range of values for that parameter from the health care payer perspective. D, Two-way sensitivity analysis illustrates how the cost-effectiveness of HB screening compared with CB screening changes when varying their screening probability parameters simultaneously, from the health care payer perspective.

The results of the 1-way sensitivity analysis are shown in Figure 1A for the societal perspective and Figure 1C for the health care payer perspective. The ICERs were most sensitive to the material and kit distribution cost for home-based screening, as well as the time, office visit, and travel costs associated with clinic-based screening. ICERs were less sensitive to sample processing costs, and lower sample processing costs favored home-based screening. Two-way sensitivity analysis presented how ICERs varied across all possible screening participation rates (Figures 1B and 1D). From the societal perspective, either clinic-based or home-based screening could be the dominant strategy depending on the combination of participation rates. However, from the health care payer perspective, the ICER generally remained above $50 and even exceeded $200 when home-based screening achieved higher effectiveness. Additional 2-way sensitivity analysis results for time and travel costs are provided in the eFigure in Supplement 1.

**Figure 2.**
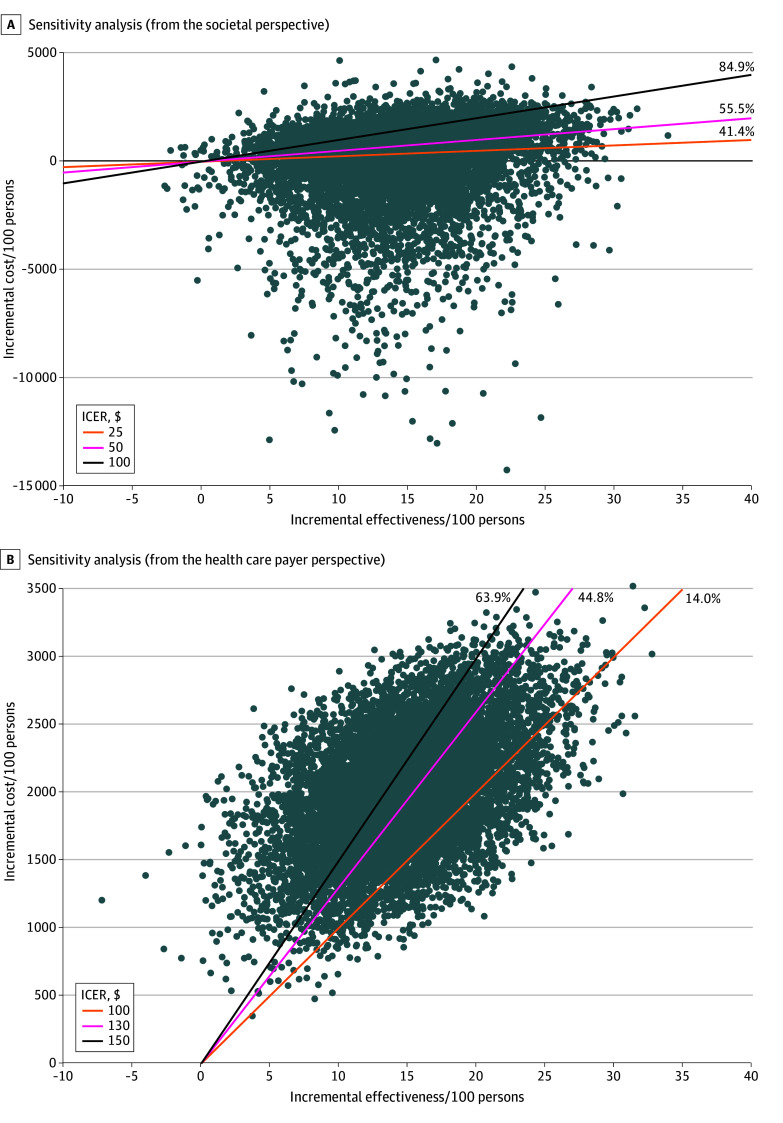
Probabilistic Sensitivity Analysis Graphs show the impact of uncertainty in model parameters on the cost-effectiveness of home-based screening compared with clinic-based screening from a societal perspective (A) and from a health care payer perspective (B). ICER indicates incremental cost-effectiveness ratio.

From the societal perspective, among 10 000 simulations, home-based screening was cost-effective in 41.4% at an ICER threshold of $25, increasing to 55.5% at a threshold of $50 and 84.9% at a threshold of $100 (Figure 2A). From the health care payer perspective, home-based screening was cost-effective in 14.0% of simulations at a $100 threshold, increasing to 44.8% at a threshold of $130 and 63.9% at a threshold of $150 (Figure 2B).

**Figure 3.**
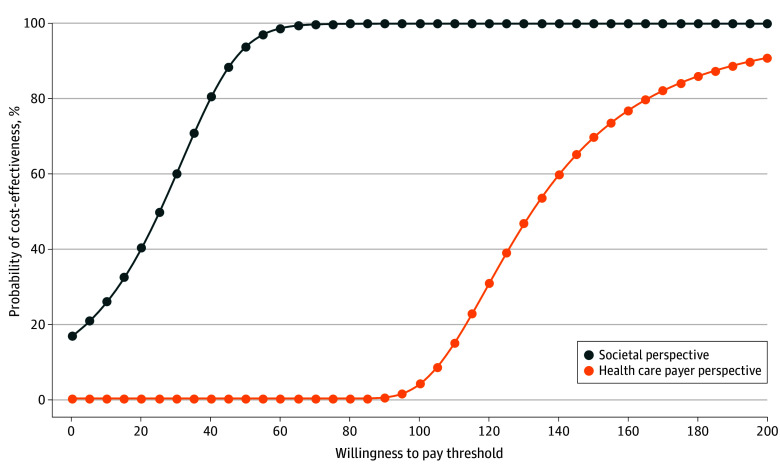
Cost-Effectiveness Acceptability Curve Graph shows the probability that home-based screening is cost-effective compared with clinic-based screening over a range of values for willingness to pay for an additional screening from both societal and health care payer perspectives.

Figure 3 presents the cost-effectiveness acceptability curves showing the likelihood that home-based screening will be cost-effective compared with clinic-based screening for a range of hypothetical decision-maker WTP values. From the societal perspective, the probability that home-based screening was cost-effective compared with clinic-based screening was 17.0% at a WTP of $0, 49.6% at a WTP of $25, 93.9% at a WTP of $50, and 99.99% at a WTP of $100. From the health care payer perspective, the probability was 3.8% at a WTP of $100, 69.6% at a WTP of $150, and 90.9% at a WTP of $200.

## Discussion

This economic evaluation study estimated the cost-effectiveness of home-based HPV self-sampling, incorporating both direct and societal costs, compared with clinic-based screening. The key finding of this study is that home-based screening resulted in an additional person being screened at an incremental cost of $25.19 from the societal perspective and $132.36 from the health care payer perspective. These findings suggest that home-based screening promises to be a cost-effective option to enhance anal cancer screening participation. In addition, the lower ICER from the societal perspective underscores the economic value of home-based screening by capturing time, travel, and productivity costs associated with clinic-based screening.

Anal cancer screening is recommended by both the HHS and the International Anal Neoplasia Society for high-risk groups (eg, MSM) with incidence rates more than 10-fold higher than the general population.^7,8^ A recent cost-effectiveness analysis conducted over the lifetime of MSM with HIV aged 35 years or older found that anal cytology every 3 years is cost-effective, with biennial screening becoming favorable (cost-effective) among newly eligible 35-year-old birth cohorts.^24^ In addition, the analysis demonstrated that HPV16/18 testing every 3 years yielded greater value and efficiency than cytology.^24^ Although the International Anal Neoplasia Society guidelines support primary high-risk HPV testing, the HHS does not recommend high-risk HPV testing without cytology among MSM with HIV because the high prevalence of high-risk HPV leads to lower specificity and positive predictive value. This aligns with recent modeling showing an unfavorable harm-to-benefit ratio and poor cost-effectiveness for this approach. However, high-risk HPV testing remains valuable as a triage strategy, particularly with genotyping for HPV16/18, consistent with HHS recommendations. HHS also specifies that only Clinical Laboratory Improvement Amendments–certified laboratories should perform anal HPV testing. Compared with clinic-based screening, home-based approaches rely critically on adequate sample collection. Previous research found comparable HPV detection rates between home-based (96.3%) and clinic-based (93.3%) screening.^17^ Together with our findings, these results support home-based high-risk HPV testing as a feasible and potentially cost-effective method to increase screening participation, and as a promising triage option with an incremental cost-effectiveness ratio of $25 from a societal perspective.

Our study also has key implications for addressing screening barriers. A recent study^25^ using nationally representative data from the Centers for Disease Control and Prevention’s Medical Monitoring Project (June 2019 to May 2020) showed that only about 5% of people with HIV, who are at the highest risk for anal cancer, had undergone anal cytology in the past year. Engagement in anal cancer screening faces several barriers, including limited health care access, low awareness, and a lack of physician recommendation. Anal cancer screening is also compounded by stigma related to the anus, receptive anal sex, SGM status, and cultural norms around masculinity.^14,26^ Home-based screening may help reduce these barriers by minimizing the need for clinic visits, decreasing indirect costs such as travel and time, and offering a more private and acceptable option for individuals who may experience stigma. Considering that nearly one-third of men indicated they would be willing to take the test if it cost up to $150 (equivalent to $213 in 2023 US dollars),^26^ the estimated cost of home-based screening at $66.97 appears favorable. The same study reported that more than 80% of the individuals are willing to take the test if it is free.^26^ Our findings suggest that home-based screening at an incremental cost of $132 per additional person screened (health care payer perspective) could be an effective strategy for overcoming cost barriers. From a societal perspective, a lower ICER of $25 highlights the impact of home-based screening on reducing indirect costs, including time and travel expenses. However, practical challenges exist for implementing mailed kit programs, including uncertain insurance coverage, the need for reliable laboratory infrastructure, and clear follow-up pathways for abnormal results. In addition, as of now, the US Food and Drug Administration has not approved self-sampling for anal cancer screening, highlighting that these approaches remain investigational and require careful implementation in research or pilot programs.

Increasing cancer screening participation remains a public health priority. A variety of strategies, including patient navigation, reminder letters, telephone calls, and mailing screening kits, have shown a median (IQR) incremental cost of $279 ($189-$528) per additional person screened for breast cancer, $110 ($45-$609) for colorectal cancer, and $315 ($189-$553) for cervical cancer.^27,28^ Given the difficulty of comparing our results with broader interventions, including navigation-based programs, and the similarities between anal and cervical cancer in natural history and risk factors, home-based cervical cancer screening studies may provide valuable insights.^29^ Several studies have shown that mailing self-sampling kits for cervical cancer increases participation.^30,31^ Two European studies reported incremental costs of approximately €63 ($74) in France^32^ and €38 to €64 ($44-$75) in Finland.^33^ In Washington State, a recent study found the ICER of a mailed HPV self-sampling intervention was $86 (95% CI, $86-$86) using the cost basis of Kaiser Permanente Washington wellness visits, and $146 (95% CI, $146-$146) using the basis of Medicare Papanicolaou test–only visits.^34^ Another study from North Carolina analyzed the cost-effectiveness of self-sampling paired with scheduled assistance and found an ICER of $284 (ICER range based on 1000 simulations, $219-$414) based on the Medicaid-state cost basis. The study concluded that higher costs may largely be due to added community outreach costs.^35^ Compared with these cervical and other cancer screening programs, the incremental cost of $132.36 (from a health care payer perspective) per additional individual screened through home-based anal cancer screening is promising from a cost-effectiveness standpoint. Lower ICER values, when accounting for patient time and travel costs—factors that home-based screening can help eliminate—highlight another potential benefit of home-based screening for anal cancer. All these studies, including ours, define participation over a relatively short term. Considering the convenience of home-based screening for SGM individuals, the long-term impact may be greater. Here, adherence to screening, including repeat and up-to-date screening rates, needs further investigation.

We extensively evaluated how varying completion rates impact the results. As participation declines, the fixed costs of home-based screening, such as kits and distribution, are amortized across fewer completions, resulting in a higher per-screen cost and higher ICERs. In contrast, clinic-based screening typically incurs costs only when the screening is completed; thus, it is less sensitive to participation rates. Only a few studies have examined participation rates in home-based screening for anal cancer. In a study where participants were asked to bring their screening kits to the clinic, 10% of participants declined, and 80% of MSM returned their kits, resulting in an overall participation rate of approximately 72%.^36^ Another study investigated the use of home-sampling kits for screening sexually transmitted infections and HIV and found that 81% of MSM found home-based sampling acceptable.^37^ However, evidence on completion rates for home-based anal cancer screening remains limited. To address this uncertainty, we modeled a full range of plausible participation scenarios. Our findings demonstrated that cost-effectiveness is sensitive to participation rates, highlighting the importance of achieving high completion rates in home-based screening programs.

### Limitations

Limitations to this study include transportation and time cost estimates based on self-reported data. These costs and ICER values, along with screening participation and other factors, may vary across target populations (eg, non-MSM with HIV or women with HIV) or the general population. This study was conducted in an urban setting where proximity to clinics may reduce structural barriers; therefore, the findings may not be generalizable to individuals living in rural areas. In rural contexts, longer travel distances, limited clinician availability, and constrained clinic capacity may reduce participation and alter the relative cost-effectiveness of screening strategies. Indeed, our sensitivity analysis suggests that home-based screening may yield higher value for individuals living farther from health care facilities, where transportation, time, and opportunity costs associated with clinic visits are higher. In addition, variation in socioeconomic status, insurance coverage, and cultural factors may influence the willingness and ability to engage in either home-based or clinic-based screening. Moreover, clinic-based screening may involve additional costs for some individuals that were not accounted for in this study, such as childcare costs during screening visits. Including such costs would likely further reduce the ICER for home-based screening. This study defines participation over a relatively short time frame. Given the convenience of home-based screening for SGM individuals, its long-term impact may be greater. Further research is warranted to assess adherence over time, including repeat and up-to-date screening rates. The analysis did not include downstream steps such as follow-up clinic visits, confirmatory testing (eg, cytology or high-resolution anoscopy), treatment of precancerous lesions, or cancer prevention. To fully evaluate the value of home-based screening, future studies should model the entire continuum of care, incorporating long-term clinical outcomes and estimating ICERs in terms of cost per life-years and quality-adjusted life-years gained. In addition, the comparative effectiveness of home-based screening with other screening modalities (eg, cytology, triage options, and cotesting) should be further investigated. Furthermore, because of ethical considerations, individuals participating in home-based screening were still asked to visit a clinic for digital anal rectal examination after they completed home-based screening, to detect and exclude those with prevalent anal cancer. However, this study does not include the cost of follow-up visits in the cost-effectiveness analysis.

## Conclusions

In conclusion, our study assessed the cost-effectiveness of home-based anal cancer screening using data from a randomized clinical trial. The economic evaluation estimated the costs and effects of both home-based and clinic-based screening, along with the uncertainty surrounding these estimates. We found that home-based screening costs $25.19 per additional individual screened from a societal perspective and $132.36 per additional individual screened from a health care payer perspective. Our sensitivity analysis showed that participation rates, kit material cost, and distribution costs are the key factors for cost-effectiveness. These findings will help decision-makers in considering the uncertainties, implementation requirements, and cost structures, and determining their WTP for a home-based anal cancer screening program.

## Notes

Supplement 1. eTable. Characteristics of participants included in the cost-effectiveness analysis eFigure. Two-way sensitivity analyses eReference

Supplement 2. Data Sharing Statement
