# Supplementary material for: Cost-Effectiveness of Home-Based Self-Sampling vs Clinician Sampling for Anal Precancer Screening
Source: JAMA Netw Open. 2026 Jan 5;9(1):e2552220. doi: 10.1001/jamanetworkopen.2025.52220 (PMC12771244; doi:10.1001/jamanetworkopen.2025.52220)
Supplement: Supplement 1. — eTable. Characteristics of participants included in the cost-effectiveness analysis eFigure. Two-way sensitivity analyses eReference [file jamanetwopen-e2552220-s001.pdf]

Supplemental Online Content

Damgacioglu H, McAuliffe TL, Ridolfi TJ, et al. Cost-effectiveness of home-based self-sampling vs clinician sampling for anal precancer screening. *JAMA Netw Open*. 2026;9(1):e2552220. doi:10.1001/jamanetworkopen.2025.52220

eTable. Characteristics of participants included in the cost-effectiveness analysis

eFigure. Two-way sensitivity analyses

eReference

This supplemental material has been provided by the authors to give readers additional information about their work.

© 2026 Damgacioglu H et al. *JAMA Network Open*.

eTable: Characteristics of participants included in the cost-effectiveness analysis. Participants were randomized to home or clinic arm in the Prevent Anal Cancer Self-Swab Study, Milwaukee, Wisconsin, 2020-2022, n (%). Additional details are available in the published study.[1]

Characteristic	Home (n=120)	Clinic (n=120)
**Age, years, median (IQR)**	46 (33–57)	45 (33–59)
**Age, years (categorical)**		
25–34	35 (29.2%)	36 (30.0%)
35–44	20 (16.7%)	23 (19.2%)
45–54	24 (20.0%)	21 (17.5%)
55–78	41 (34.2%)	40 (33.3%)
**Gender identity**		
Man	115 (95.8%)	112 (93.3%)
Transgender, nonbinary, or other	5 (4.2%)	8 (6.7%)
**Race/ethnicity**		
White, non-Hispanic	76 (63.3%)	82 (68.3%)
Black, non-Hispanic	26 (21.7%)	19 (15.8%)
Hispanic	15 (12.5%)	16 (13.3%)
Other, non-Hispanic	2 (1.7%)	3 (2.5%)
Missing	1 (0.8%)	0
**Education, years**		
≤12	12 (10.0%)	18 (15.0%)
13–15	34 (28.3%)	30 (25.0%)
16	22 (18.3%)	22 (18.3%)
>16	51 (42.5%)	50 (41.7%)
Missing	1 (0.8%)	0
**Health insurance**		
No	6 (5.0%)	8 (6.7%)
Yes	114 (95.0%)	110 (91.7%)
Missing	0	2 (1.7%)
**HIV**		
Negative	82 (68.3%)	93 (77.5%)
Positive	38 (31.7%)	27 (22.5%)

© 2026 Damgacioglu H et al. *JAMA Network Open*.

eFigure. Two-way sensitivity analyses

A) Two-way sensitivity analysis shows how the cost-effectiveness of home-based screening compared with clinic-based screening changes when varying the clinic time parameters simultaneously, from the societal perspective. Clinic times for clinic-based screening were analyzed from 10 minutes to 1 hour, while sample collection time for home-based screening ranged from 0 to 20 minutes. B) Two-way sensitivity analysis shows how the cost-effectiveness of home-based screening compared with clinic-based screening changes when varying the travel time parameters simultaneously, from the societal perspective. Travel times for clinic-based screening were analyzed from 10 minutes to 1 hour, while travel times for home-based screening ranged from 0 to 20 minutes. C) Two-way sensitivity analysis shows how the cost-effectiveness of home-based screening compared with clinic-based screening changes when varying the travel cost parameters simultaneously, from the societal perspective. Travel costs for clinic-based screening were analyzed from $0 to $25, while travel costs for home-based screening ranged from $0 to $10.

A.

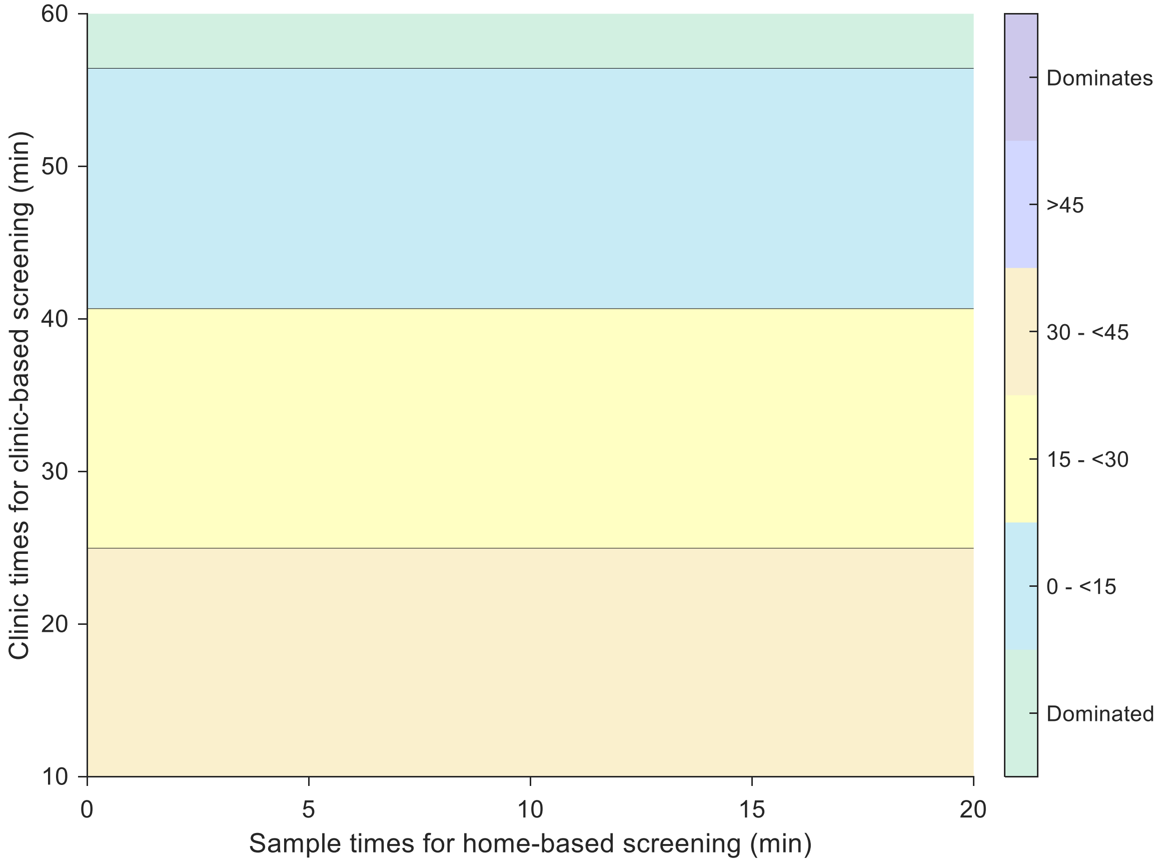

© 2026 Damgacioglu H et al. *JAMA Network Open*.

B.

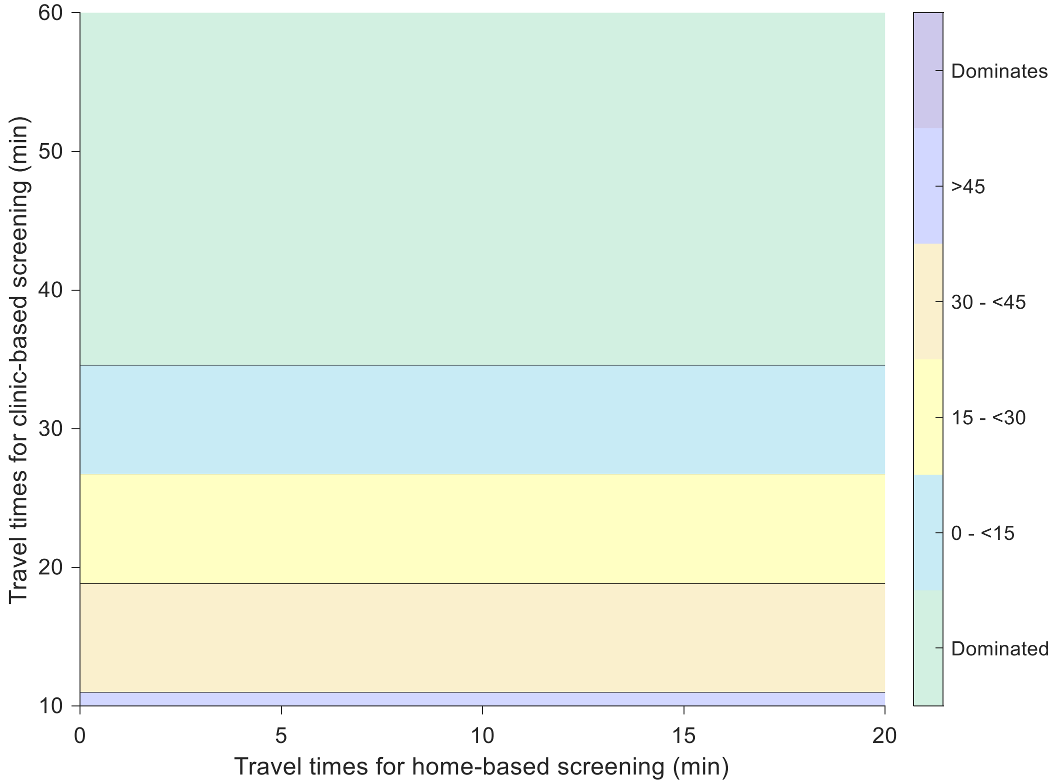

C.

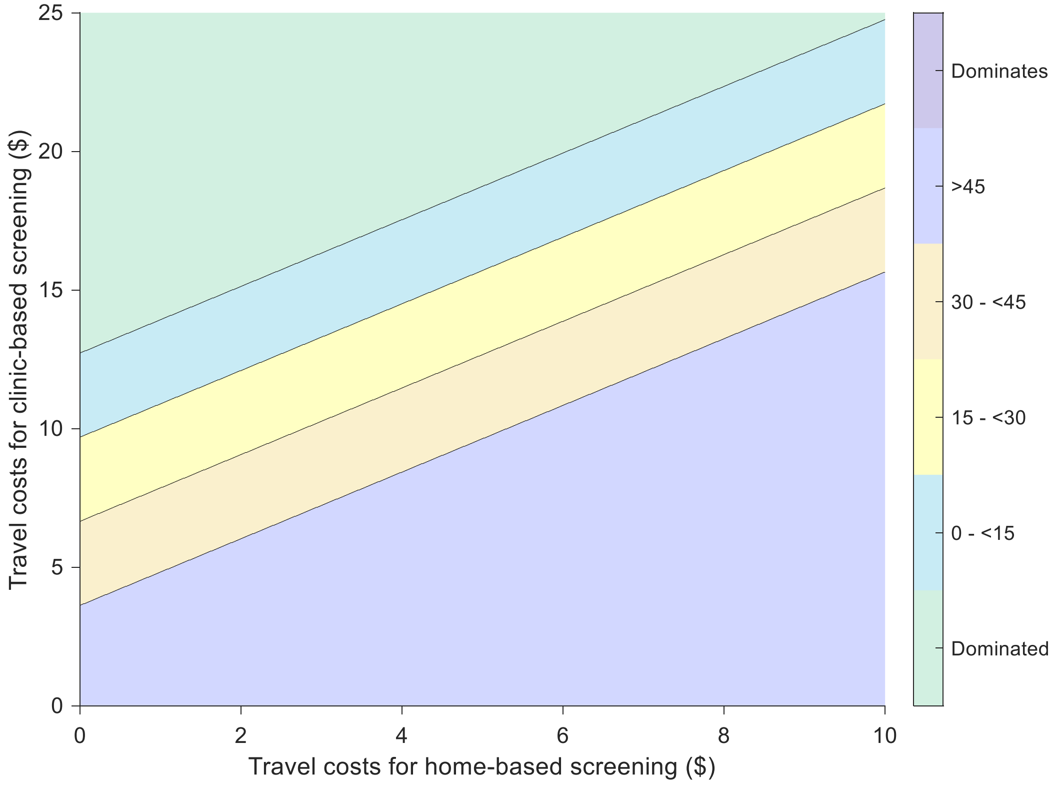

© 2026 Damgacioglu H et al. *JAMA Network Open*.

eReference

1. Nyitray AG, Nitkowski J, McAuliffe TL, et al. Home-based self-sampling vs clinician sampling for anal precancer screening: The Prevent Anal Cancer Self-Swab Study. Int J Cancer. Aug 15 2023;153(4):843-853. doi:10.1002/ijc.34553

© 2026 Damgacioglu H et al. *JAMA Network Open*.
