# Supplementary material for: Cost-Effectiveness of Home-Based Self-Sampling vs Clinician Sampling for Anal Precancer Screening
Source: JAMA Netw Open. 2026 Jan 5;9(1):e2552220. doi: 10.1001/jamanetworkopen.2025.52220 (PMC12771244; doi:10.1001/jamanetworkopen.2025.52220)
Supplement: Supplement 2. — Data Sharing Statement [file jamanetwopen-e2552220-s002.pdf]

Data Sharing Statement

Damgacioglu. Cost-Effectiveness of Home-Based Self-Sampling vs Clinician Sampling for Anal Precancer Screening. *JAMA Netw Open*. Published January 05, 2026.
doi:10.1001/jamanetworkopen.2025.52220

Data
Data available: No

Additional Information
Explanation for why data not available: The study used clinical trial data.
